# Cultural competence and associated factors among nurses working in public health institutions in the Assosa zone, Benishangul Gumuz regional state, Ethiopia, 2022

**DOI:** 10.1186/s12912-023-01488-2

**Published:** 2023-10-09

**Authors:** Mulualem Birhanu, Asmamaw Getnet, Girma Alem

**Affiliations:** 1 Department of Nursing, College of Health Science, Assosa University, Assosa, Ethiopia; 2 Department of Nursing, College of Health Science, Debermarkos University, Debermarkos, Ethiopia

**Keywords:** Ethiopia, Cultural competence, Nurses

## Abstract

**Background:**

Cultural competence is one of the principal foundations of clinical nursing. In Ethiopia, nurses in nursing care still focus more on physical needs, the healing process, and treatment and less on the cultural aspects of the patient.

**Objective:**

This study aims to assess the cultural competence and associated factors among nurses working in public health institutions found in the Assosa Zone, West Ethiopia, in 2022.

**Methods:**

An institution-based cross-sectional study design was conducted on 362 nurses who were selected by simple random sampling. Data was collected using a structured, self-administered English version of the Nurse Cultural Competence Scale Questionnaire for Nurses. The data were entered into Epi Data version 3.1 and exported to SPSS version 25. Linear regression analysis was used to identify factors statistically significantly associated with the cultural competence of nurses at a *p*-value < 0.05.

**Result:**

Overall The mean score of cultural competence of participants was 113 (CI, 111.7–115.7), with a mean item score of 3.2 (CI, 3.15–3.26).

Nurse-to-patient ratio (B;.93, CI;.59_1.3), experience with previously working in a primary hospital than the current health institution (B; -11.1, CI; -18_-4.2), and experience with previously working in a health center than the current health institution (B; -11.5, C;-18.5_-4.8), being diploma education level (B; -23.2, CL;-32_-14.8), being BSC education level (B;-20.3, CI;-28_-12.3), and the presence of a feedback system in a health facility (B; 13.5, CI; 9.5_17.5) were identified as predicted factors of cultural competences.

**Conclusion:**

The overall mean score of the cultural competencies of the participants was moderate. To improve the cultural competence of nurses, it is typically necessary to provide educational opportunities to raise their educational level and establish a feedback system in all health institutions across the nation.

## Introduction

Cultural competence is one of the principal foundations of clinical nursing. It is both a process and a result of the integration of the skills and knowledge we acquire throughout our personal and professional lives, as well as the continuing improvements we make [1, 2].

Twenty million nurses worldwide deliver excellent, life-changing healthcare to an increasingly diverse population. However, globally the major cause of health disparities is the existence of cultural diversity, which leads to poorer patient outcomes and lower satisfaction with care [3, 4].

Racial, gender, and ethnic inequities as well as cultural competency are ongoing problems in the current healthcare practice. Many members of racial and ethnic minority groups are at a greater risk of contracting COVID-19 and dying from it as a result of long-standing systemic health and socioeconomic disparities [5, 6].

Even after adjusting for biological and social factors, research in a few European countries has indicated that women from minority ethnic groups, particularly those with Turkish ancestry, had lower pregnancy outcomes than native women [7].

Unintentional errors in the healthcare system include failure to plan actions to be completed as intended, using the incorrect plan to achieve the objective, and deviating from the course of care in a way that might damage patients. More than 250,000 people die from these health-related mistakes every year, making them the third most common cause of mortality in the US [8, 9]. A systematic scoping review conducted in Canada, New Zealand, Australia, and the United States showed that 31% of the participants had poor culturally competent nursing care [10]. According to a study done on Sudanese nurses, 58.7% of the participants got low scores for cultural competency [11].

There are no surveys or systemic reviews that showed the cultural competence level of Ethiopian nurses, however, a study conducted among nurses working in Addis Ababa tertiary hospitals showed that the cultural competence level of participants was low to moderate [12]. Also, another study conducted in southwest Ethiopia was Lowe level (1.95) [13].

Language barriers, cultural differences, discrimination, and the physicians' lack of knowledge or skill are the most frequent obstacles that patients who are culturally and linguistically diverse (CLDP) confront while trying to receive healthcare services [14–16].

A systematic review of a review conducted in Australia identified five categories of intervention that were included: the use of community-based bi-lingual health workers; providing cultural competency training for health workers; using interpreter service; using multimedia and culturally sensitive videos to promote health, and establishing community point-of-care services for culturally appropriate service’s [17].

In Ethiopia, there is no established evidence quantifying the impact of culturally incongruent nursing care on the outcome of patients. But, the study conducted in Indonesia showed that in patients who received nursing care from a nurse who had cultural competence, satisfaction with his service was 5.2 times higher compared to patients who were cared for by nurses who had less Competence [11].

Despite the existence of a multicultural society that needs healthcare providers to be culturally competent, in Ethiopia, there is still little research on the cultural competence of nurses. Studies conducted in Ethiopia are mostly in referral hospitals and the study populations for all studies are nurses who are working in tertiary hospitals [9, 12]. Therefore This study fills the above gap by including nurses who are working in health centers, primary hospitals, and general hospitals. Furthermore, this was the first study in this study area.

## Materials and methods

### Study area, study design, study setting, and sample

Benishangul/Gumuz Region has internal boundaries with Amhara and Oromia Regions and is located on Ethiopia's western border with Sudan. The Grand Ethiopian Renaissance Dam (GERD) is located in this region. The major ethnic groups living in this region are Amhara (25.41%), Berta (21.69%), Gumuz (20.88%), Oromo (13.55%), Shinasha (7.73%), and Agaw-Awi (4.22%). The main languages are Berta (25.15%), Amharic (22.46%), Gumuz (20.59%), Oromo (17.69%), Shinasha (4.58%), and Awngi (4.01%), just like other regional states; however, different ethnic groups also live in the Region. Concerning religion, 44.7% were Orthodox Christians, 33.3% Muslim, 13.53% were Protestant, and 7.09% practiced traditional beliefs as of Wednesday 2/23/2022 11:35 PM Google finds. Assosa zone is located 667 km the West of Addis Ababa. It has a population of 342,287; male 188,258 and female 154,029. The zone has 7 woredas (districts) and 72 kebeles (villages) [18]. In this zone, there are 31 health centers and 2 governmental hospitals. The total number of nurses working in this facility is 512, which I get from BGRHB. The study was conducted on June 1–30/2022.

An institution-based cross-sectional study design was conducted. The study was conducted from Jun1-30/2022. The study was conducted among all public health institutions found in the Assosa zone, Benishangul Gumuz Regional State, and North West Ethiopia. The source population of the study was all nurses who were working in public health institutions in the Assosa zone. Considering that the Study population was nurses who were working in public health facilities and available during the data collection period and Nurses who were ill during the data collection period were excluded from the study.

### Sample size determination

The sample size was calculated by using a formula for a single population mean with a consideration of the following assumptions: Level of confidence = 95% The margin of error = 0.07, calculated by taking the standard deviation from a previous study [12].

$$n= \frac{{\left(z\alpha /2\right)2 \mathrm{x} \left(\sigma \right)}^{2}}{d2}= \frac{\left(1.96\right)2*\left(0.65\right)2=329}{{\left(0.07\right)}^{2}}$$

so, the final sample size by adding 10%( 33) of the non-response rate was 362.

### Sampling procedure

**Fig. 1:**
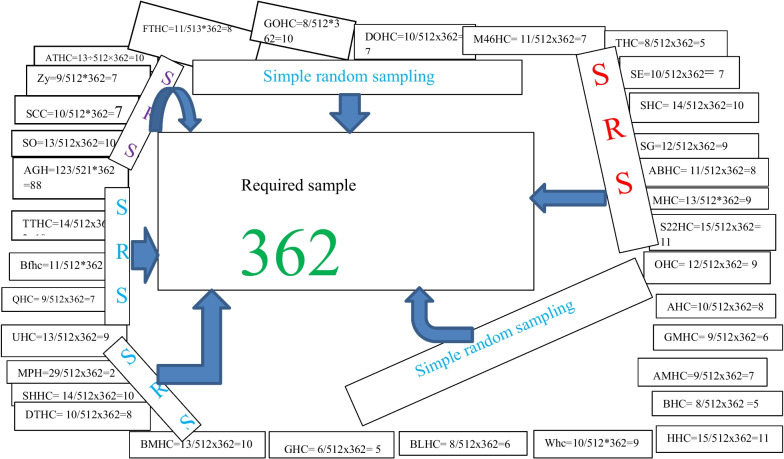
Sampling procedure. First, the appropriate participant number was distributed to every two hospitals and 31 health centers proportionally to the number of nurses employed in each hospital and health center utilized by using formula $\frac{{\varvec{n}}{\varvec{o}}}{{\varvec{N}}}\times {\varvec{n}}$,no; total population in each unit, N; total source populations, n; required sample size. The Participants were selected from a sample frame by using a simple random sampling technique in each hospital and health center as proportionally allocated

The appropriate participant number was distributed to each hospital and health center proportionally to the number of nurses employed in each hospital and health center utilized. The Participants were selected from a sample frame by using a simple random sampling technique in each health facility as proportionally allocated (Fig. 1).

### Variables of the study

#### Dependent variable

Level of Cultural competence.

#### Independent variables

##### Socio-demographic factors

Sex, age, ethnicity, marital status, religion, education level in a year, and work experience in a year.

##### Institutional factors

The presence of feedback systems, nurse-to-patient ratio, and level of health institution served previously.

##### Cultural experience-related factors

Ability to speak another Ethiopian language in addition to Amharic and English, Caring for culturally diverse patients, Frequency of caring for culturally diverse patients, Experience of work other than their current institution, and Experience in taking training related to cultural nursing care.

#### Operational definition

##### Level of Cultural competence

Total score ranges from 36 to 180 with scores near to higher scores indicative of higher cultural competence and near to lowest scores indicative of cultural incompetency [19].

For descriptive purposes, the overall cultural competence item mean score and each subscale item mean score were categorized:

The low (mean item score = 1–1.80).

The low to moderate (mean item score = 1.81–2.6).

The moderate (mean item score = 2.61–3.4).

High (mean item score ≥ 3.41) [9].

##### Patients from different Ethnic and cultural backgrounds

Patients who grew up in Ethiopia but have different ethnic backgrounds (Amhara, Gurage, Oromo, Gumuz, etc.) or the same ethnic background but come from other geographic areas and different cultural backgrounds [12].

##### Cultural awareness

Is a process of in-depth Self-examination of one's claim social foundation, values, stereotypes, biases, and practices and recognizing their impact when interacting with people from diverse cultures [2, 20].

##### Cultural knowledge

Is the process of learning about the similarities and differences of various cultures and comprehending how this knowledge affects one's practices and values [2, 20].

##### Cultural sensitivity

is a process in which nurses view their clients as partners in during appropriate care, and treat their patients as unique individuals with unique needs [2].

##### Cultural skill-

Is being able to gather information about a person's culture and traditions, doing a physical checkup based on their culture, and identifying their main health issue right away [20, 21].

### Data collection tools

Socio-demographic variables were collected using a structured, self-administered English version of a study-specific questionnaire. Nurses' cultural competence was measured using a structured, self-administered English version of the Nurse Cultural Competence Scale Questionnaire for Nurses (NCCSQ), which was adapted by a previous researcher in Ethiopia [12]. Each item uses a five-point Likert scale to measure the participant's response: 1 = totally disagree, 2 = 25% agree, 3 = 50% agree, 4 = 75% agree, and 5 = 100% agree. In this study, Cronbach’s alpha for this tool was 0.933.

### Data collection procedure

Ten (10) data collectors with a BSc degree in nursing were chosen to collect data, and five (5) supervisors with MSC were also chosen to supervise the day-to-day activities during data collection. Participants were identified from each health facility by a simple random sampling technique using the lottery method and approached privately on arrival, without disturbing the routine working activity in the hospital. The objective of the study was explained to them using study-specific information and enough time was given to make an independent decision to participate in the study.

### Data quality assurance

The training was given to both data collectors and supervisors on the instrument, data collection procedure, and the ethical principles of confidentiality. A pretest was conducted on 20 nurses working in a mend hospital before the actual data collection period to determine the tool's reliability. Besides all this, the data were checked for completeness during data collection, entry, and compilation before the analysis was made.

### Data processing and analysis

The data were coded, entered, and cleaned using Epi-data version 3.1 and exported to SPSS Version 25. The characteristics of the study population were described using descriptive statistics, including mean, standard deviations, and frequency, and described in the form of texts and tables.

After assumptions related to linear regression models were checked, linear regression analysis was carried out. Linearity: Scatter plots demonstrate that the linearity assumption between the independent variables and dependent variables had been met, Normality: p-p plot showed normal distribution of the residuals (dots lie closer to the diagonal line, Multi-collinearity: tolerance value range 0.21–1 and VIF ranges 1_4.7, No auto-correlation: Durbin-Watson test = 2.02, Homoscedasticity: scatter plot showed that the spread of the residuals was fairly constant, which suggests the assumption of homoscedasticity was successful, and No influential outliers: no cooks distance over one.

Simple linear regression was done to select the factors candidates for multiple linear regression at a *p*-value < 0.25 and finally, in multiple linear regression, statistical significance was accepted at a *p*-value < 0.05.

The single composite variable (level of cultural competence of nurses) was determined by simply averaging the original variables' scores, such as cultural knowledge score, cultural awareness score, cultural sensitivity score, and cultural skill score.

## Result

### Socio-demographic characteristics

A total of 362 questionnaires were distributed, and all of them were correctly filled out and returned, giving a response rate of 100%. The mean age of the participant was 32 ± 5 years ranging from 23–48 years. Of the 362 respondents, about 212 (58.6%) of them were male. About 107 (29.6%) of them were Oromo by ethnicity. Nearly half of 168(46.8) of the participants were followers of the Orthodox religion.

Concerning marital status the majority 236(65%) of participants were unmarried. Regarding the level of education, more than half of 201(55.5%) participants were Bachelor's degree holders. The mean duration of work experience was 6 ± 3 years ranging from 1 to 17 years (Table 1).

**Table 1:** Socio-demographic characteristics of nurses working in Public Health institutions found in Assosa zone in Benishangul gumuze, Ethiopia 2022

*Variable*	*Categories*	*Frequency*	*Percentages*	*M* ± *SD*
Gender	Male	212	58.6%	
	Female	150	41.4%	
Religion	Orthodox	168	46.4%	
	Muslim	101	27.9%	
	Catholic	1	0.3%	
	Protestant	84	23.2%	
	others specific	8	2.2%	
Ethnicity	Amhara	99	27.3%	
	Berta	43	11.9%	
	Oromo	107	29.6%	
	Shinasha	89	24.6%	
	Other	24	6.6%	
Level of education	Diploma	145	40.1%	
	BSC degree	201	55.5%	
	Master’s degree	16	4.4%	
Marital status	Married	126	34.8%	
	Unmarried	236	65.2%	
Age				32 ± 5
Work experience				6 ± 3

### Institutional and cultural experience-related factors

About 309 (85.4%) participants can speak another Ethiopian language in addition to Amharic and English. 342 (94.5%) participants had experience in caring for patients from culturally and ethnically diverse with 244(71.3%) of them having an opportunity to care for these patients almost every day.

Concerning working in any health institution other than their current health institution more than half 204(56.4%) of participants had work experience other than their current health institution with 84(41.0%) of them working in a health center. Concerning the presence of a feedback system in the institution majority305 (79%) of the respondent revealed the presence of a feedback system in their institutions. On average daily, each participant provides care for 11 ± 5 patients ranging from 2 to 25 (Table 2).

**Table 2:** Institutional and Cultural experience-related factors of nurses working in a public health institution in Assosa zone, Benishangul Gumuz, Ethiopian (*n* = 362)

*Variable*	*Categories*	*Frequency*	*Percentage*	*M* ± *SD*
Ability to speak any language(s) other than Amharic and English	Yes	309	85.4%	
	No	53	14.6%	
Experience with caring for people who are culturally and ethically different from you	Yes	342	94.5%	
	No	20	5.5%	
Frequency of caring, diversified patients	almost everyday	244	71.3%	
	1or2 times week	60	17.5%	
	1or2times a month	6	1.8%	
	Several times a year	32	9.4%	
Worked in any health institution other than your current hospital	Yes	204	56.4%	
	No	158	43.6%	
Level of health institution (*n* = 205)	General Hospital	23	11.2%	
	primary hospital	71	34.6%	
	health center	84	41.0%	
	health post	27	13.2%	
Presence of a feedback system in a health institution	Yes	287	79.3	
	No	75	20.7	
Nurses to patient ratio				11 ± 5

*M* Mean, *SD* Standard deviation

### Cultural competence of participants

The mean score of cultural competence of participants was 113.7 ± 19 (95% CI, 111.7–2115.7), ranging from 62 to 162. The mean scores of the participant's cultural awareness subscale were 24.2 ± 5.2 ranging from 11 to 35. The mean score of the participant's cultural knowledge subscale was 33.6 ± 6.6 ranging from 17 to 50. The cultural sensitivity mean score was 30.5 ± 5.5 ranging from 15 to 45. The cultural skill subscale mean score was 25.4 ± 6.5 ranging from 11 to 50.

Based on the overall mean item score of cultural competencies and the mean item score of each subscale, the total overall cultural competence mean item score was 3.2 ± 0.53 ranging from 1.7 to 4.5. The participant's cultural awareness subscale was high (M ± SD = 3.46 ± 0.73), whereas, the cultural knowledge (M ± SD = 3.36 ± 0.65), and cultural sensitivity (MSD = 3.40.6) subscales were moderate. Despite that, the subscale of cultural skills was low to moderate (M ± SD = 2.5 ± 0.65) (Table 3).

**Table 3:** Each Subscale and the total scores of the CCQN scale of mean scores of nurses working in a public health institution in Assosa zone, Benishangul Gumuz, Ethiopia (*n* = 362)

*Variable*	*Domain score*		*Mean item score of the domain*		*Level of cultural competence*
	***Range***	***M* ± *SD***	***M* ± *SD***	***Range***	
Cultural awareness	11–35	24.2 ± 5.2	3.5 ± .74	1.6–5	High level
Cultural knowledge	17–50	33.6 ± 6.6	3.4 ± .66	1.7–5	Moderate level
Cultural sensitivity	15–45	30.5 ± 5.5	3.4 ± .6	1.7–5	Moderate level
Cultural skill	11–50	25.4 ± 6.5	2.5 ± .65	1.1–5	Low to moderate
TotalCCQN scale score	62–162	113.7 ± 19	3.2 ± .53	1.7–4.5	Moderate level

*M* Mean, *SD* Standard deviation

### Factors influencing cultural competence

In the Simple linear regression model variables such as gender, age, Work-experience in years, nurse-to-patient ratio, level of health institution worked other than current health institutions, educational level, Experience with caring for patients culturally and ethnically different, and Presence of feedback system in a health institution were associated with cultural competence at *p*-value < 0.25 and they transferred to multiple linear regression.

In multiple linear regressions, nurse-to-patient ratio, experience with working in other health facilities than the current health facility, level of health facility previously worked than the current health facility, level of education, and presence of feedback in the health institutions were identified as factors influencing the cultural competence of nurses at a *p*-value < 0.05.

A significant *F* –test equation was found (*F (*12,349) = 19.66, *p*- value = 000 with an adjusted *R*^2^ = 0.383, 38.3% of the variation in cultural competence of nurses was explained by independent variable in the model (Table 4).

**Table 4:** Model summary

*R*	*R Square*	*Adjusted Square*	*SE Estimate*	*P*
.635	.403	.383	14.96	.000

### Interpretation of the results

The presence of feedback systems in health institutions increases the mean score of cultural competence of nurses by 13.9 units compared to health institutions that hadn’t feedback mechanism (B; 13.9, CI; 9.98–17.9).

Nurse to patients ratio was a predictor of the cultural competence of nurses implies that the number of patients they gave the care to increase by one resulting in a 0.929 unit increment in a mean score of cultural competence (B; 0.929, CI; 0.597–1.26).

Nurses who had a diploma level of education were associated with a reduction of 23.3 units in a mean score of cultural competence as compared to nurses who had a master's level of education (B; -23.3, CI;-31.6, -14.8).

Nurses who had a BSc degree level of education were associated with a reduction of 20.2 unit in a mean score of cultural competence as compared to nurses who had a master level of education(B; -20.2, CI; -28.4,-12.239).

Compared to nurses who were not worked in other health facilities than the current health facility, those who worked in other health facilities than the current health facility were associated with an increment of 13 units in a mean score of cultural competencies (B; 13, CI; 6.2–9.816).

Experiences with working in primary hospitals other than current health facilities were associated with a reduction of 11.1 units in a mean score of cultural competence compared to those who had work experiences in general hospitals than current health facilities (B;-11.1, CI; -17.9,-4.16).

Experiences with working in health centers other than current health facility associated with a reduction of 11.6 units in a mean score of cultural competence compared to those who had work experiences in general hospitals than current health facilities (B;-11.6, CI, -18.5,-4.7) (Table 5).

**Table 5:** Multiple linear regression factors with their unstandardized and standardized coefficients unit change in cultural competence mean scores of nurses working in a public health institution in Assosa zone, Benishangul Gumuz, Ethiopia (*n* = 362)

**Variable**	**Unstandardized Coefficients(B)**	**Std. Error**	**Standardized Coefficients(ß)**	***P***	95.0% CI	
					LB	UB
(Constant)	95.1	9.1		.00	77	113
Age	.405	.241	.11	.09	-.1	.9
Gender: Female (*reference group: male)*	-.452	1.651	-.012	.7	-3.7	3
Work experience	.529	.36	.09	.14	-.2	1.2
Presence of a feedback system in a health facility(*reference groups: no)*	13.5	2.1	.29	.00	9.5	16
Nurse-to-patient ratio	.93	.17	.23	.00	.59	1.3
Diploma level education*(Reference groups master level)*	-23.2	4.3	-.6	.00	-32	-15
BSC level of education (*Reference groups: a master level)*	-20.3	4.1	-.5	.00	-28	-12
Experience with caring for patients culturally and ethnically different( *Reference groups: no*)	.8	3.6	.01	.83	-6.2	7.7
Working in other health institutions than current health institution*(reference group: no)*	13.2	3.5	.34	.00	6.2	19.8
previously working in a primary hospital *(reference group previously worked in general hospital)*	-11.1	3.5	-.23	.002	-18	-4.2
previously working in a health center *(reference group: previously worked in general hospital)*	-11.6	3.5	-.26	.01	-18.5	-4.8
Previously working in a health post *(reference group: previously worked in general hospital)*	-8.1	4.3	-.11	.06	-16.4	.32

*N.B. CI* Confidence interval, *LB* Lower boundary, *UB* Upper boundary

## Discussion

The overall mean score of cultural competencies of the participants was moderate. Moreover, the Nurse-to-patient ratio, experience with previously working in a primary hospital than the current health institution, and experience with previously working in a health center than the current health institution, being diploma education level, being BSC education level, and the presence of a feedback system in a health facility were identified as predicted factors of cultural competences.

This finding is in line with the cross-sectional sturdy conducted in Ethiopia [9]. It is also consistent with a study conducted in China [22].

The mean score of cultural competence of nurses in the current study was higher than the studies conducted in South Africa [23] and South Asian countries [24] with the mean scores of participants were low. The possible reason could be the time gap of the study conducted, health system differences, and tool differences where they used IAPCC- R, which has 25 items with a four-point Likert scale, but the current study used CCQN which has 36 items with a five-point Likert scale.

The result of the current study was lower than the study done in Korea which revealed that the mean score of participants was high [25]. This might be due to the sample size (*n* = 108) and tool (the tool was Caffrey Cultural Competence in Healthcare Scale with 41 items) difference.

The participants scored lowest in the subscale of cultural skill, this is consistent with the findings of the studies conducted in Ethiopia and South Africa [12, 23], The possible reason could be that the participants were insufficiently equipped with health or illness-related cultural knowledge and didn’t have training related to cultural care nursing [26]. The highest score in the cultural competence subscale was cultural awareness, which was consistent with the previous study conducted in Texas, Japan, and Taiwan [27–29].

As the current study showed that a diploma and BSc level of education is associated with a reduction of 23.3 and 20.2 units in the mean score of cultural competence as compared to being master’s level education respectively. The possible reason could be nurses who have a master's level of education have taken transcultural nursing care as part of the chapter on the foundation of nursing in the post-graduate program, are more oriented to acquiring advanced training, and are engaged in better self-learning generally when compared to nurses who have diplomas and degree level of education. This result was consistent with the studies conducted in Ethiopia, China, Italy, and Saudi Arabia [9, 22, 26, 30]. This result underlines the importance of specific cultural competence educational interventions for nurses with less than master-level preparation.

The current study revealed that the presence of a feedback mechanism in health institutions was positively associated with the cultural competence of nurses. This finding was supported by the report of a study conducted in a similar country and a systematic review conducted across Europe [9, 31]. The possible reason could be feedback is intended to enhance professional performance and thereby improve the quality of health care and patient safety [31].

The result of this study indicates that experiences with working in primary hospitals other than current health facilities were associated with a reduction of 11.1 units in a mean score of cultural competence compared to those who had work experiences in general hospitals than current health facilities. Also, it shows that experiences with working in health centers other than the current health facility associated with a reduction of 11.6 units in a mean score of cultural competence compared to those who had work experiences in general hospitals than current health facilities This finding was supported by the studies conducted in Ethiopia [32] and Saudi Arabia [26].

The possible explanation might be the workforce in a general hospital is more diverse than the workforce in primary and health centers which aligned with diversifying the workforce of healthcare providers increasing the diversity and cultural competence of the healthcare workforce [33]. In addition, only one general hospital was found and it gives service to people coming from different communities with different cultural backgrounds. This provides a great opportunity to provide service to people from different linguistic and cultural backgrounds. Indirectly enhances their cultural competence.

The nurse-to-patient ratio was also a significant predictor of the cultural competence of nurses in this research. A possible explanation could be nurses providing care frequently for diverse patient combines their knowledge and skills with awareness, curiosity, and sensitivity about their patients’ cultural beliefs [1]. This issue needed further research to verify the association between the nurse-to-patient ratio and the cultural competence of nurses. Since no previous study examined the association between the nurse-to-patient ratio and the cultural competence of nurses.

### Implication for nursing practices

This is a calling alarm to all healthcare managers and leaders to change the current practice and provide higher quality patient care by developing and integrating nursing care plans that take into account the patient's culture and being open and compassionate.

### Limitations of the study

Self-administered questionnaires might encourage people to answer based on social desirability rather than real belief and lead to the tendency of participants to answer the question in the way they perceive and understand the questions.

Cross-sectional studies are by their nature limited to a single point in time; therefore, it is not possible to generalize the study findings beyond that.

## Conclusions and recommendation

This study reveals a moderate level of cultural competencies among nurses working in a public health facility in the Assosa zone. Nurse-to-patient ratio, working in other health facilities than the current health facility, level of health facility previously worked than the current health facility, level of education, and presence of feedback in health institutions were identified as predictor factors of cultural competence of nurses**.**

Therefore nurses need to attempt to develop their cultural competence by reading any available books related to cultural nursing care, and healthcare organizations need to provide them with adequate support for cultural competence development by sufficiently equipping them with health or illness-related cultural knowledge, preparing and motivating staff to attend seminars and conference on cultural competence, And providing training related to cultural care nursing.

## Data Availability

All data generated or analyzed during this study are included in this published article.
